# mRNA and tRNA modification states influence ribosome speed and frame maintenance during poly(lysine) peptide synthesis

**DOI:** 10.1016/j.jbc.2022.102039

**Published:** 2022-05-17

**Authors:** Tyler J. Smith, Mehmet Tardu, Hem Raj Khatri, Kristin S. Koutmou

**Affiliations:** Department of Chemistry, University of Michigan, Ann Arbor, Michigan, USA

**Keywords:** ribosome, RNA modification, translation stalling, peptide-mediated stalling, reading frame maintenance, aatRNAs, aminoacyl-tRNA, eTLC, electrophoretic TLC, IC, initiation complex, m^6^A, N6-methyladenosine, TC, ternary complex

## Abstract

Ribosome speed is dictated by multiple factors including substrate availability, cellular conditions, and product (peptide) formation. Translation slows during the synthesis of cationic peptide sequences, potentially influencing the expression of thousands of proteins. Available evidence suggests that ionic interactions between positively charged nascent peptides and the negatively charged ribosome exit tunnel impede translation. However, this hypothesis was difficult to test directly because of inability to decouple the contributions of amino acid charge from mRNA sequence and tRNA identity/abundance in cells. Furthermore, it is unclear if other components of the translation system central to ribosome function (*e.g.*, RNA modification) influence the speed and accuracy of positively charged peptide synthesis. In this study, we used a fully reconstituted *Escherichia coli* translation system to evaluate the effects of peptide charge, mRNA sequence, and RNA modification status on the translation of lysine-rich peptides. Comparison of translation reactions on poly(lysine)-encoding mRNAs conducted with either Lys-tRNA^Lys^ or Val-tRNA^Lys^ reveals that that amino acid charge, while important, only partially accounts for slowed translation on these transcripts. We further find that in addition to peptide charge, mRNA sequence and both tRNA and mRNA modification status influence the rates of amino acid addition and the ribosome’s ability to maintain frame (instead of entering the −2, −1, and +1 frames) during poly(lysine) peptide synthesis. Our observations lead us to expand the model for explaining how the ribosome slows during poly(lysine) peptide synthesis and suggest that posttranscriptional RNA modifications can provide cells a mechanism to precisely control ribosome movements along an mRNA.

Translation of the nucleic acid code into protein is catalyzed by the ribosome. During this process, ribosomes use mRNAs as molecular maps to direct the programmed assembly of amino acids into polypeptides. In *Escherichia coli* growing polypeptide chains are extended by an average of 4 to 22 amino acids per second, though the rate of individual amino acid incorporation by the ribosome is not always uniform (1, 2, 3). Heterogeneity in peptide elongation rates is caused by a number of factors, including substrate (*e.g.*, mRNA, aminoacyl-tRNAs [aatRNAs], translation factors) availability and modification status, and the formation of stable interactions between the growing polypeptide chain and the ribosome machinery (4, 5). Although translation initiation rates are responsible for controlling the rate of protein expression in many circumstances, situations that alter polypeptide elongation rates can change protein levels, protein folding, and mRNA stability to ultimately impact cellular health and fitness (6, 7, 8).

Contacts between the ribosome and its nascent peptide products are receiving growing recognition for their role in translationally controlling protein expression (9). The interactions involving the ribosome and positively charged peptides present a classic example of this phenomenon. There is overwhelming evidence that translating the cationic peptide sequences commonly present in proteins slows the ribosome (10, 11). In humans, there are over 60,000 examples of proteins containing four or more consecutive basic amino acids, suggesting that the synthesis of positively charged peptides contributes to the posttranscriptional control of a significant fraction of the proteome (Tables S1 and S2). The observation that ribosomes slow while linking iterated positively charged amino acids has long been attributed to the formation of strong ionic interactions between cationic peptides and the anionic ribosome peptide exit channel (12). However, several recent reports demonstrate that the ribosome produces different amounts of protein from mRNAs possessing synonymous codons that encode identical positively charged poly(lysine) and poly(arginine) peptides (13, 14, 15). Ionic interactions alone cannot explain these findings, suggesting that additional factors in the translation system also contribute to modulating ribosome speed during the synthesis of cationic peptides.

Multiple codons instruct the ribosome to add the positively charged amino acids lysine (AAA and AAG) and arginine (AGA, AGG, and CGN [N=U, C, A,G]). The ability of individual arginine codons to differentially impact protein expression largely depends on the availability of tRNA isoacceptors possessing appropriate anticodon sequences. Some isoacceptors are less abundant, and translation along mRNA sequences containing multiple codons corresponding to these rare tRNAs can slow sufficiently to trigger cellular mechanisms that rescue stalled ribosomes (16, 17). The cause of differential protein expression from the two lysine codons appears to differ from that of arginine codons. In the case of lysine, less protein is produced from mRNAs containing consecutive AAA codons than those with consecutive AAG codons in both eukaryotic and bacterial cells (13, 14, 18, 19). However, differences in substrate tRNA levels are unlikely to account for these codon-specific observations because AAA and AAG are decoded by a single tRNA^Lys^ in at least one of the species (*E. coli*) where codon-dependent differences in poly(lysine) protein output have been observed. Furthermore, in addition to reducing the rate of protein synthesis, under some conditions the presence of two or more AAA codons in a row can promote an unusual ribosome movement termed “ribosome sliding” (13). During sliding, the ribosome loses reading frame and shifts along an mRNA. The ribosome has been captured moving backward by 1 to 3 nucleotides while translating iterated AAA codons (13, 14), changing the identity of the peptide being made. These movements activate cotranslational surveillance mechanisms that target the translated mRNA and resulting peptide products for degradation (18). Ribosome sliding differs from other noncanonical ribosome movements, which place the ribosome at a single, discrete location on an mRNA and can produce stable products (20, 21).These data suggest that the influence of mRNA and tRNA sequences on the translation of poly(lysine) peptide regions warrant further examination.

Here, we use a reconstituted *E. coli* translation system to deconvolute the contributions of peptide, mRNA sequence, and RNA (mRNA and tRNA) modification to both the speed of amino acid addition and ribosome frame maintenance during the translation of iterated lysine codons. We chose to investigate the role of RNA modifications in addition to peptide and mRNA sequence because these common chemical changes to nucleosides can alter the hydrogen-bonding interactions between tRNAs and mRNAs used by the ribosome to ensure the faithful and rapid translation of the genetic code into protein (22). Our findings expand the biochemical framework for understanding the contributions of individual components of the translation system to ribosome stalling during cationic peptide synthesis. We demonstrate that in addition to peptide charge, mRNA sequence, along with mRNA and tRNA modification status, are important determinants of ribosome speed during poly(lysine) translation. Additionally, we developed a minimal kinetic mechanism for ribosome sliding on iterated AAA codons, in which the ribosome moves along an mRNA in the 3′ direction one nucleotide at a time, until it can bind an available cognate aatRNA and resume “normal” translation in a different frame. Much like ribosome speed, this series of one nucleotide ribosome movements is controlled not only by peptide charge but also by posttranscriptional modifications to tRNA^Lys^ and mRNA (N6-methyladenosine [m^6^A]). While it has been known for decades that tRNA anticodon stem-loop modifications can influence ribosome movements, these data provide the first evidence that mRNA modifications also have the power to impact ribosome reading frame maintenance (23). Our work presents a molecular level rationalization for how seemingly small changes in the translational machinery (*e.g.*, synonymous codon substitution and single posttranscriptional modifications) can result in different protein production outcomes.

## Results

### Ribosomes move backward one nucleotide at a time on poly(A) sequences

**Figure 1:**
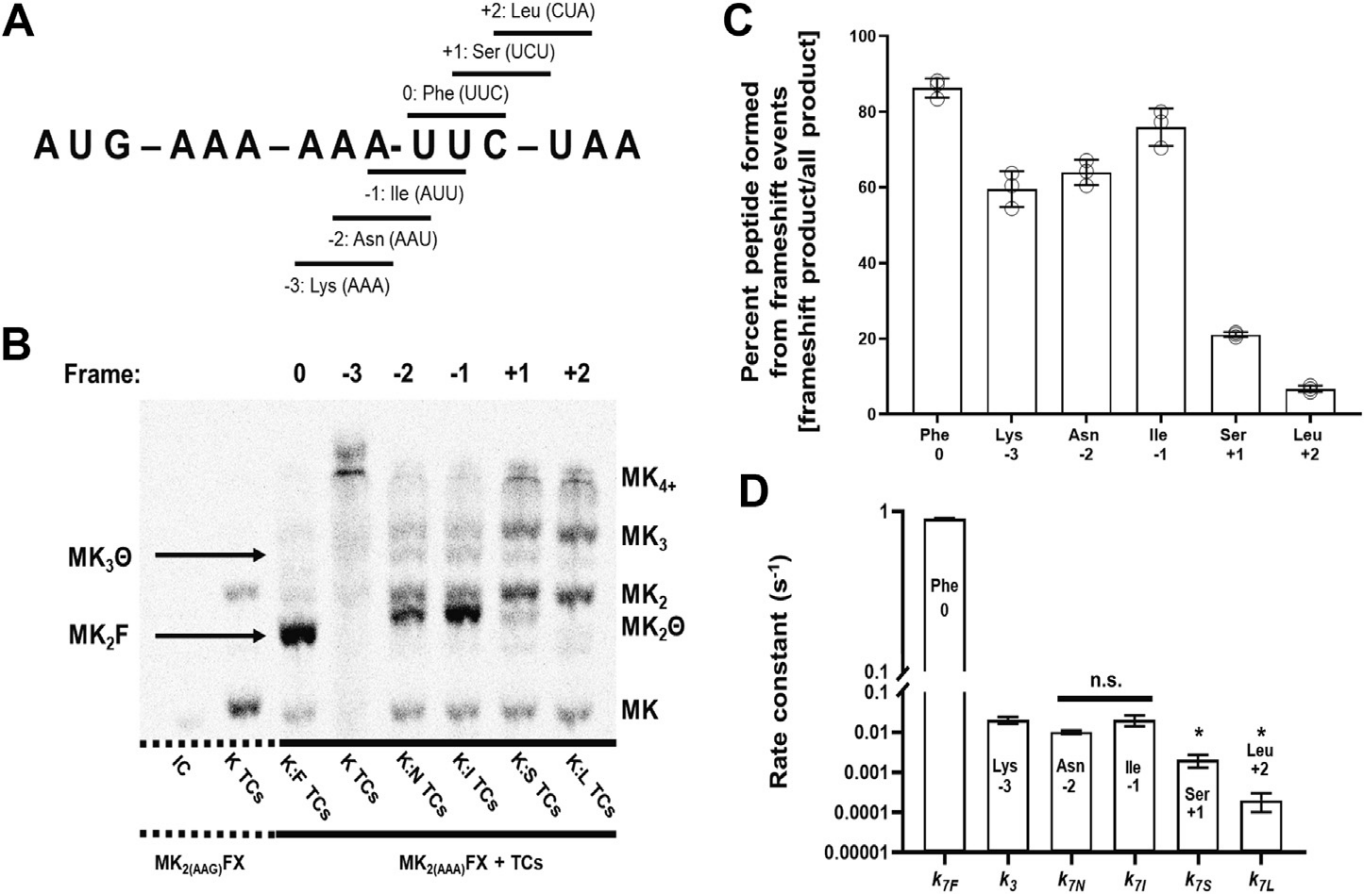
**The ribosome moves into multiple frames on poly(A) in absence of next cognate aatRNA**^**aa**^**.***A*, possible ribosome-sliding events on MK_2(AAA)_FX mRNA result in new discrete codons positioned in the A-site, allowing for decoding and accommodation of noncoded aatRNA^aa^. *B*, phosphorimage of a eTLC displaying the products of 20 min end-point reactions investigating frameshifting during ribosome sliding on MK_2(AAA)_FX or MK_2(AAG)_FX mRNA incubated with Lys-tRNA^Lys^ and various aatRNA^aa^ TCs. Lanes indicate the aatRNA^aa^ TCs used in each reaction, with the numbers correlating to the amino acid identity indicated in panel (*A*) (*e.g.*, −2 indicates reaction performed with Lys-tRNA^Lys^ and Asn-tRNA^Asn^). Θ indicates the amino acid that would be added upon successful frameshift and incorporation of aatRNA^aa^ as indicated per lane and in panel (*A*) (*e.g.*, bands for MK_2_Θ and MK_3_Θ in −2 lane correspond to the peptides MK_2_N and MK_3_N, respectively). *C*, percent of peptide product formed due to sliding/frameshifting compared to total peptide synthesized during translation assays, with frame and third amino acid added as signified. Error bars represent SD. *D*, rate constants for frameshift events during ribosome sliding on MK_2(AAA)_FX mRNA using Lys-tRNA^Lys^ and aatRNA^aa^ TCs as specified with frame (panel *A*), as defined by the proposed mechanism in Figure 2. Error bars represent SD. There is no difference in the rate constants for amino acid addition in the −1 (Ile; *k_7I_*), −2 (Asn; *k_7N_*), and −3 (Lys; *k_3_*) frames. For +1 (Ser; *k*_*7S*_) and +2 (Leu; *k*_*7L*_) rate constants, a ‘ ∗ ’ represented a significant alteration with a *p*-value <0.05 using a unpaired student *t* test when compared to the −3 rate constant (Lys; *k*_*3*_). aatRNAs, aminoacyl-tRNA; eTLC, electrophoretic TLC.

There are multiple ways to envision how ribosome sliding on consecutive AAA codons could be achieved. For example, the ribosome might hop directly into the −1 and −3 frames, “scan” along an mRNA until it reaches a specific, desired frame, or make series of discrete one nucleotide frameshifts (13, 14, 24, 25). We developed a kinetic framework to distinguish between these possibilities and describe how the ribosome moves during sliding using a fully reconstituted *in vitro* translation system (26). To accomplish this, we first identified the reading frames the ribosome enters during translation along an mRNA with a AUG-AAA-AAA-UUC-UAA (MK_2(AAA)_FX; X=stop codon) coding sequence (Fig. 1*A*). In these assays, 70 nM of *E. coli* 70S ribosome initiation complexes (ICs) containing ^35^S-labeled formylmethionine-tRNA^fMet^ bound to an AUG in the P site and an AAA codon in the A site were reacted with saturating concentrations of two ternary complexes (TCs, 10–30 μM; aatRNA^aa^⋅EF-Tu⋅GTP) formed with elongation factor-Tu (EF-Tu) as well as cognate Lys-tRNA^Lys^ and individual aatRNA^aa^ species capable of reacting in each of the reading frames that the ribosome could inhabit on our MK_2(AAA)_FX mRNA (−2 frame, Asn-tRNA^Asn^; −1 frame, Ile-tRNA^Ile^; 0 frame, Phe-tRNA^Phe^; +1 frame, Ser-tRNA^Ser^; and +2 frame, Leu-tRNA^Leu^) (Fig. 1*A*). These reactions were conducted in the presence of 0 to 12 μM of elongation factor G GTPase bound to GTP (EF-G⋅GTP) at 37 °C. The reactants (^f^Met), programmed peptides (MK, MK_2_, MK_2_F), and peptide products resulting from the ribosome sliding (MK_2_I, MK_2_N, MK_2_S, MK_2_L, MK_3_, and MK_4+_) were visualized by electrophoretic TLC (eTLC; Fig. 1*B*). In our assays, the ribosome generated products in five different reading frames (0, −1, −2, −3, and +1; Fig. 1, *A*–*C*). We find that movement of the ribosome into non-0 frames is EF-G dependent, much like canonical-1 frameshifting ribosome movements (Fig. S1) (24, 27, 28, 29).

**Figure 2:**
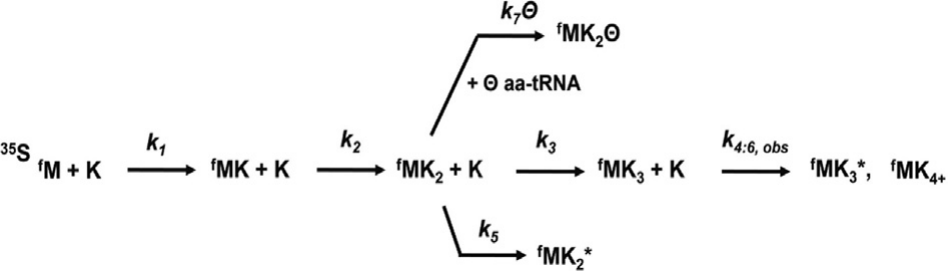
**Proposed general scheme for frameshift events during ribosome sliding on poly(A).** In the case where available aatRNAs are incorporated *via* frameshift, denoted by *Θ*, this scheme describes subsequent amino acid additions by a ribosome translating on a poly(A) containing mRNA—as displayed in Fig. 1. The scheme contains parameters obtainable from the experiments presented here: dipeptide formation (*k*_*1*_), tripeptide formation (*k*_*2*_), ribosome sliding, and frameshift events (*k*_*3*_, first −3 sliding/frameshift event generating tetrapeptide [MK_3_]; *k*_*5*_, first sliding/frameshift event resulting in unproductive ribosome(s); *k*_*7*_*Θ*, first sliding/frameshift of ribosome moving into new coding frame [MK_2_Θ]), and secondary/tertiary sliding events capable of occurring following first sliding/frameshift event (*k*_*4:6,obs*_). aatRNAs, aminoacyl-tRNA.

After establishing which frames the ribosome inhabits during sliding, we measured the extent of amino acid incorporation in each of these frames at discrete time points (0–1200 s, Figs. 1*C* and S2). These data were used to develop a minimal kinetic mechanism for ribosome sliding by globally fitting our experimental observations with KinTek Explorer (KinTek Corporation; https://kintekexplorer.com/) (Fig. S2). We examined a series of possible mechanisms (Fig. S3) and selected the model that best fit our data to ascertain the rate constants for each step in the mechanism. Our fits indicate that the ribosome undergoes a series of progressive −1 nucleotide movements from the 0-frame into the −1, −2, and −3 frames during ribosome sliding (Figs. 2 and S4). The rate constants for amino acid addition in the −1, −2, and −3 frames are relatively uniform (k_7_ values range between 0.01–0.02 s^−1^) (Fig. 1*D* and Table 1). Furthermore, a subset of ribosomes (∼10%) appear unable to extend the growing polypeptide following each progressive −1 nucleotide ribosome movement (Figs. 1*B* and 2 (k_5_, k_4:6,obs_), Figs. S3 and S5*B*). While we do observe a small amount of product formation in the +1 frame, both the endpoint and rate constant for this reaction are diminished relative to the same values for the ribosome reacting in the −1, −2, and −3 frames (Fig. 1, *C* and *D*). These observations lead us to propose a model for ribosome sliding in which a small (<5% of ribosomes) can undergo a +1 frameshift, while most ribosomes move in the 3′ direction by one nucleotide a time until they enter a reading frame that can react with an available aatRNA species (Fig. 2).

**Table 1:** Rate constants for frameshift and amino acid addition during ribosome sliding

Frameshift position	0 frame	−2 frame	−1 frame	+1 frame	+2 frame
tRNA TCs	Lys + Phe	Lys + Asn	Lys + Ile	Lys + Ser	Lys + Leu
Rate constants (s^−1^)					
*k*_*1*_	11.7 ± 0.01	13.1 ± 0.01	15.3 ± 0.8	12.7 ± 0.7	13.3 ± 0.7
*k*_*2*_	1.5 ± 0.002	1.6 ± 0.002	1.4 ± 0.2	1.5 ± 0.15	1.2 ± 0.3
*k*_*3*_	-	0.005 ± 0.0002	0.008 ± 0.0015	0.008 ± 0.001	0.02 ± 0.004
*k*_*4:6,obs*_	-	0.006 ± 0.002	0.003 ± 0.001	0.007 ± 0.003	0.002 ± 0.009
*k*_*5*_	-	0.0008 ± 0.00004	0.0007 ± 0.0003	0.002 ± 0.0005	0.017 ± 0.009
*k*_*7*_	0.9 ± 0.0007	0.01 ± 0.0009	0.02 ± 0.006	0.002 ± 0.0007	0.0002 ± 0.0001

### tRNA^Lys^ modifications moderate ribosome sliding during poly-lysine synthesis

**Figure 3:**
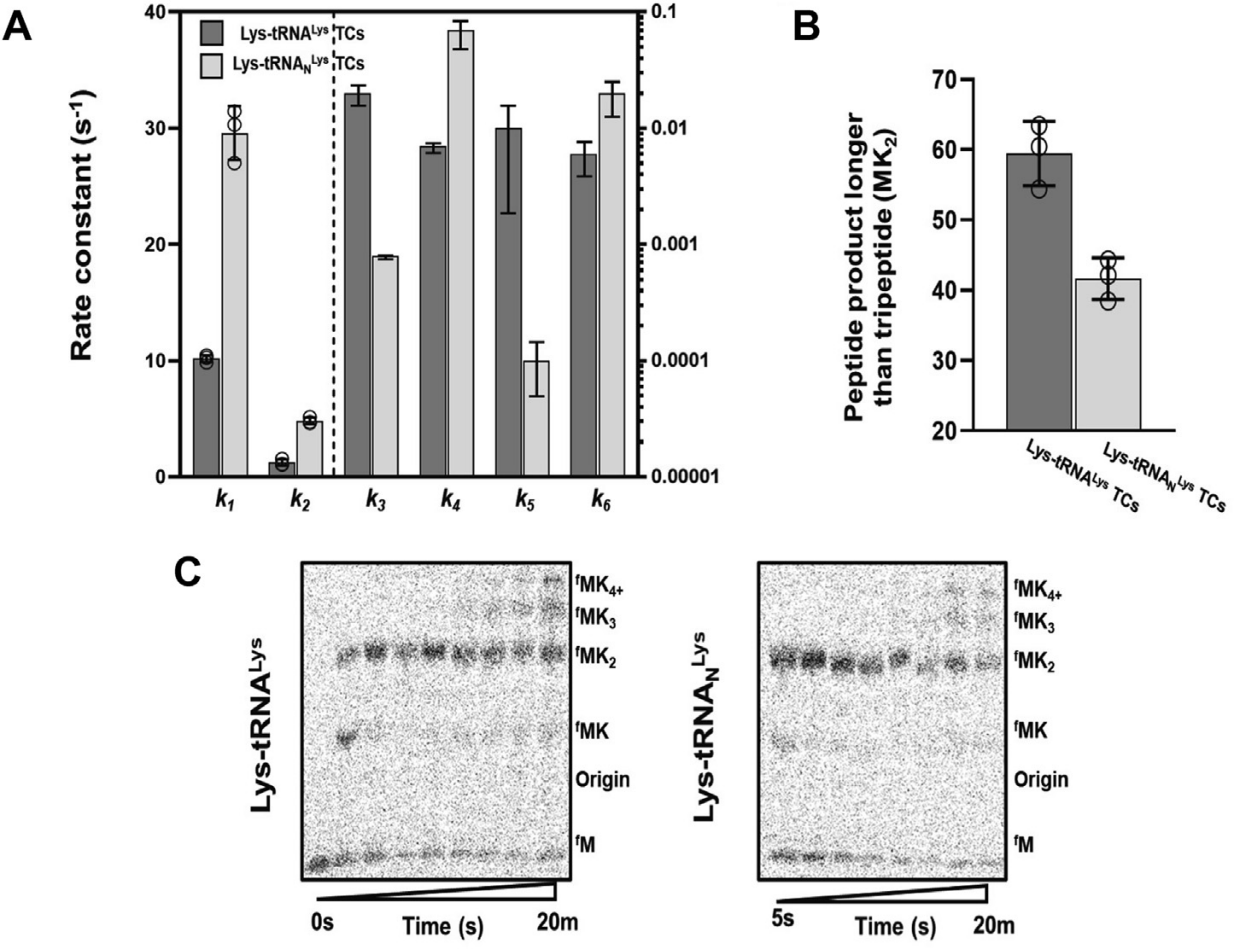
**Modifications on tRNA**^**Lys**^**regulate extent of frame loss on poly(A) mRNA.***A*, rate constants for ribosome sliding on MK_2(AAA)_FX mRNA using either Lys-tRNA^Lys^ or Lys-tRNA_N_^Lys^ TCs, as defined by the proposed mechanism in Fig. S3*A*. Error bars represent SD. *B*, percent of total peptide formed as a result of ribosome sliding (peptides longer than MK_2_ tripeptide) on MK_2(AAA)_FX mRNA after 20 min in translation assays conducted with Lys-tRNA^Lys^ or Lys-tRNA_N_^Lys^ TCs. Error bars represent SD. *C*, phosphorimage eTLCs of of ribosome sliding over time on MK_2(AAA)_FX mRNA in translation reactions perfomred with either Lys-tRNA^Lys^ TCs (*left*) or Lys-tRNA_N_^Lys^ TCs (*right*). eTLC, electrophoretic TLC; TC, ternary complex.

Native tRNAs possess posttranscriptional chemical modifications essential to their stability, structure, and function (30, 31). Modifications located in tRNA anticodon stem loops have the capacity to modulate −1 and +1 ribosomal frameshifts and enhance ribosome reading frame maintenance (32, 33). Since the tRNA^Lys^ N6-threonylcarbamoyladenosine (t_6_A_37_) and 5-methoxycarbonylmethyl2-thiouridine [mcm_5_S_2_U_34_] modifications in yeast tRNA^Lys,UUU^ influence tRNA decoding, we speculated that analogous *E. coli* tRNA^Lys^ modifications, such as 5-methylaminocarbonylmethyluridine (mnm_5_s_2_U_34_), might suppress ribosome sliding and enhance poly(lysine) translation (34, 35). To test this idea, we compared the rate constants for lysine addition during the translation of AUG-AAA-AAA-UUC-UAA [MK_2(AAA)_FX] and AUG-AAG-AAG-UUC-UAA [MK_2(AAG)_FX] messages using saturating levels (20–30 μM) of unmodified T7 transcribed Lys-tRNA^Lys^ and natively modified Lys-tRNA_N_^Lys^ purified from *E. coli* cells (Figs. 3*A* and S6). We find that the rate constants for programmed MK and MK_2_ peptide formation are twofold to fourfold faster when ribosome complexes are reacted with TCs containing modified Lys-tRNA_N_^Lys^ than with unmodified Lys-tRNA^Lys^. These moderate enhancements in lysine addition rate constants are observed when either AAG or AAA containing mRNAs are translated (Table 2). The modifications have a larger role on frame maintenance than on programmed lysine addition; the rate constants for forming ribosome sliding products (MK_3_ and MK_4+_) on AAA codons are decreased by up to 25-fold when natively modified Lys-tRNA_N_^Lys^ is used (Fig. 3*B*). Despite the slowed formation of these sliding products, the percentage of peptides that are eventually extended and generated sliding products is only slightly reduced by the inclusion of modifications on tRNA^Lys^ (Lys-tRNA^Lys^ = 60% ± 3% *versus* Lys-tRNA_N_^Lys^ = 40% ± 6%) (Figs. 3, *B* and *C* and S6). Our data suggest that tRNA_N_^Lys^ modifications likely limit the extent of ribosome sliding in cells.

**Table 2:** Rate constants for lysine addition during ribosome sliding

mRNA construct	MK_2(AAA)_FX		MK_2(AAG)_FX	
tRNA TCs	Lys-tRNA^Lys^	Lys-tRNA_N_^Lys^	Lys-tRNA^Lys^	Lys-tRNA_N_^Lys^
Rate constants (s^−1^)				
*k*_*1*_	10.2 ± 0.3	28.9 ± 1.9	2.7 ± 0.5	12.7 ± 0.4
*k*_*2*_	1.2 ± 0.4	4.8 ± 0.4	2.1 ± 0.7	4.4 ± 0.3
*k*_*3*_	0.02 ± 0.004	0.0008 ± 0.000008	0.006 ± 0.002	0.002 ± 0.001
*k*_*4*_*(or k*_*4,obs*_*)*	0.007 ± 0.001	0.07 ± 0.02	0.0004 ± 0.0001	0.00009 ± 0.00002
*k*_*5*_	0.01 ± 0.008	0.0001 ± 0.00005	0.001 ± 0.0001	0.001 ± 0.0002
*k*_*6*_	0.006 ± 0.002	0.02 ± 0.007	-	-

### Ribosomes slow and slide when synthesizing poly(valine) peptides from lysine-encoding mRNAs

**Figure 4:**
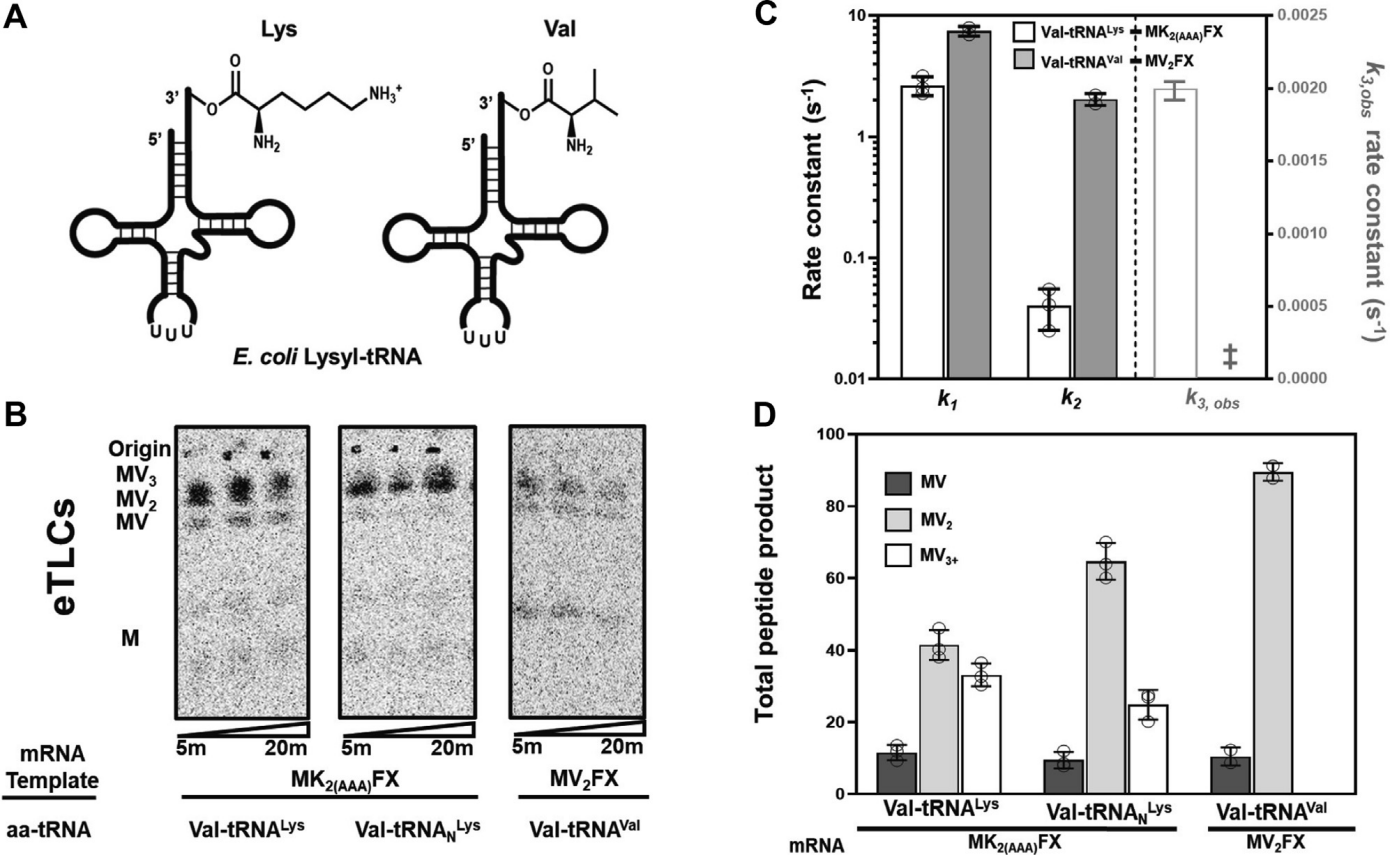
**Neutral amino acid and tRNA pairing effect ribosome sliding on poly(A) mRNA.***A*, lysyl *E. coli* tRNA_UUU_ can be acylated with positively charged lysine or misacylated with neutral charge valine by the dFx flexizyme. Flexizyme charged tRNAs used in our studies assesing influence of amino acid charge on lysine addition during translation are displayed. *B*, phosphorimages of eTLCs displaying amino acid addition and ribosome sliding on mRNAs. Translation reactions were performed with either Val-tRNA^Lys^ (*left*) or Val-tRNA_N_^Lys^ TCs (*middle*) on MK_2(AAA)_FX mRNA or Val-tRNA^Val^ (transcribed, *right*) on MV_2_FX mRNA. *C*, rate constants for amino acid addition on MK_2(AAA)_FX mRNA using Val-tRNA^Lys^ and MV_2_FX mRNA using Val-tRNA^Val^ TCs as defined by the proposed mechanism in Fig. S3*A*. The *k*_*3,obs*_ rate constant is presented on the right *y*-axis, and no rate constant was obtainable for peptide synthesis on MV_2_FX mRNA (‡) as no sliding is observed in these assays. Error bars represent SD. *D*, percent of peptide products formed during assays on MK_2(AAA)_FX or MV_2_FX mRNAs after 20 min in translation assays conducted with Val-tRNA^Lys^, Val-tRNA_N_^Lys^, or Val-tRNA^Val^ TCs as shown in panel (*B*). Error bars represent SD. eTLC, electrophoretic TLC; TC, ternary complex.

Regardless of tRNA^Lys^ modification status, we observed that after the first lysine is added into a peptide, the rate constants for adding subsequent lysines on AAA codons are reduced (Table 2 and Fig. 3*A*). This is consistent with a large body of evidence from cellular reporter and ribosome profiling studies indicating that the translation of iterated positive charges slows the ribosome (36). However, the observation that different poly(lysine) encoding mRNA sequences differentially impact translation leads us to wonder if mRNA sequence, and therefore also structure, contribute to ribosome slowing and during poly(lysine) translation (13, 14). To deconvolute the effects of peptide charge from mRNA sequence, we mischarged unmodified tRNA^Lys^ and natively modified tRNA_N_^Lys^ sequences with the small nonpolar amino acid valine (Val-tRNA^Lys^, Val-tRNA_N_^Lys^) (Fig. 4*A*). Misacylation was accomplished using a small RNA microhelix (flexizyme) capable of attaching an esterified aminoacid acyl donor to virtually any tRNA of interest (37). ICs containing mRNAs encoding consecutive lysines (AUG-AAA-AAA-UUC-UAA and AUG-AAG-AAG-UUC-UAA) were reacted with TCs possessing mischarged tRNAs (Val-tRNA^Lys^⋅EF-Tu⋅GTP, Val-tRNA_N_^Lys^⋅EF-Tu⋅GTP). Because the translation factor EF-Tu selects for correct tRNA and aminoacyl donor pairings, we titrated EF-Tu with each aatRNA to ensure saturating conditions for incorporating these species (Fig. S7) (38, 39). Concurrent control assays with TCs containing Val-tRNA^Val^ and ICs formed on an mRNA encoding consecutive valines (AUG-GUG-GUG-UUC-UAA) were also performed. The rate constants for Met-Val dipeptide and tripeptide, MV and MV_2_, formation are three-fold and five-fold slower (respectively) on AUG-AAA-AAA-UUC-UAA [MK_2(AAA)_FX] mRNA than AUG-GUG-GUG-UUC-UAA GUG [MV_2_FX] mRNA regardless of the modification status of tRNA^Lys^ (Fig. 4*C* and Table S3). In contrast, the rate constants for synthesizing MV and MV_2_ on AUG-AAG-AAG-UUC-UAA [MK_2(AAG)_FX] mRNA are reduced by less than two-fold relative to (MV_2_FX) mRNA when modified Val-tRNA_N_^Lys^ is included in the translation reaction. However, when unmodified Val-tRNA^Lys^ is used instead, the ability of the ribosome to add Val to a growing polypeptide on an AAG codon is dramatically slowed, and the rate constants for MV and MV_2_ synthesis are diminished by >1000-fold (Fig. S8 and Table S3).

In addition to impacting ribosome speed, our investigations with Val-tRNA^Lys^ and Val-tRNA_N_^Lys^ revealed that peptide charge also contributes to ribosome frame maintenance on lysine-encoding messages. We find that extended MV_3+_ peptides, analogous to the MK_3+_ peptides made during ribosome sliding (Fig. 4*B*), are generated from the AAA but not AAG or GUG containing messages. While unprogrammed MV_3+_ peptides can still be generated on consecutive AAA codons, the incorporation of additional valines is ∼10-fold slower than unprogrammed lysine addition on the same message. Our findings suggest that peptide charge and mRNA sequence make independent contributions to ribosome speed and frame maintenance during poly(lysine) peptide synthesis.

### m^6^A mRNA modifications suppress sliding on consecutive AAA codons in a position dependent manner

**Figure 5:**
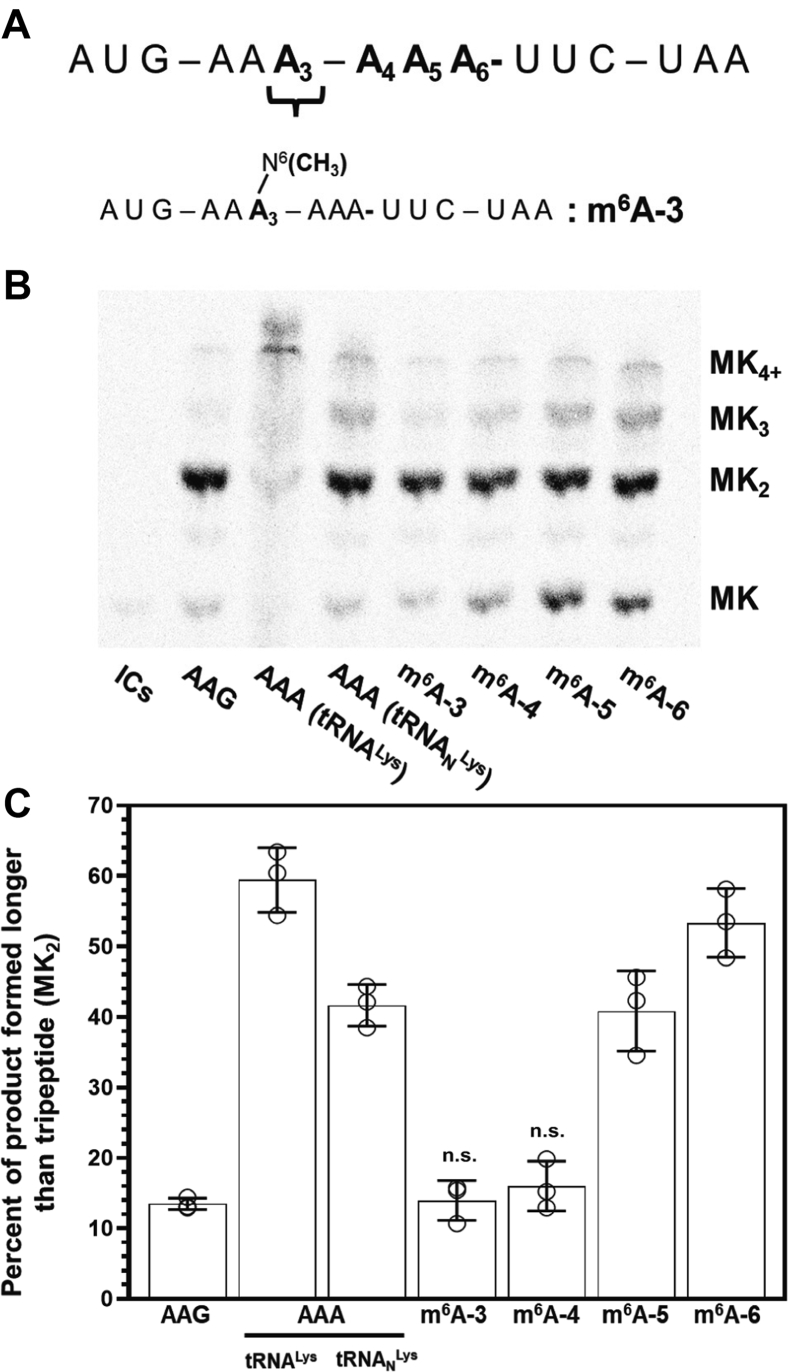
**m**^**6**^**A modification to single nucleotides in poly(A) modulate ribosome frame loss.***A*, MK_2(AAA)_FX mRNA was chemically modified with a single m^6^A on one of the six consecutive A’s. Specifically at positions A_3_, A_4_, A_5_, and A_6_ with position 3, m^6^A-3, shown. *B*, phosphorimage eTLC displaying the products of 20 min translation on MK_2(AAA)_FX transcripts harboring a single m^6^A modification at nucleotide specified. *C*, percent of total peptide formed as a result of ribosome sliding (longer than MK_2_ tri-peptide) on MK_2(AAA)_FX mRNAs that were either unmodified or harboring a single m^6^A modification after 20 min of translation using Lys-tRNA_N_^Lys^ TCs. Error bars represent SD. There is no significant difference observed for peptide product formed on messages containing m^6^A at either position A_3_ or A_4_ using Lys-tRNA_N_^Lys^ TCs when compared to peptide product formed on MK_2(AAG)_FX using Lys-tRNA_N_^Lys^ TCs when using an unpaired student *t* test. eTLC, electrophoretic TLC; TC, ternary complex.

Our data indicate that iterated AAA, but not AAG, lysine-encoding codons promote a series of consecutive −1 movements by the ribosome (ribosome sliding). We hypothesized that poly(A) regions might form a unique structure within the ribosome mRNA channel that promotes these loss of frame events. Recent cryo-EM structures of the yeast ribosome translating an mRNA sequence with six consecutive A nucleosides support this idea, revealing that stacked A’s adopt a single-stranded helix in the ribosome decoding center (40, 41). In these structures, three A’s are positioned in the mRNA A site, where they form a helical stack with residues in the 18S rRNA (41). To test the possibility that such a helical structure might enhance frame loss on poly(A) sequences, we performed translation assays on AUG-AAA-AAA-UUC-UAA [MK_2(AAA)_FX] messages with various A-nucleosides substituted with m^6^A (Fig. 5*A*) to perturb the structure of this poly(A) helix. We selected m^6^A as a probe because it has been shown to change RNA structure and dynamics (42, 43, 44). We find that when m^6^A is positioned in the middle of a six consecutive A nucleosides (at the third and fourth adenosine in the message), where they presumably could disrupt helix formation, very little extended peptide product is formed (Fig. 5, *B* and *C* and Table S4). In contrast, when the m^6^A is positioned at the fifth or sixth adenosine in the poly(A) sequence, sliding levels are comparable to those on an unmodified message (Fig. 5*C*). We examined the location of m^6^A in two available datasets that mapped m^6^A transcriptome wide to begin evaluating if our observation that m^6^A can promote frame maintenance could have relevance in endogenous A-rich mRNA coding sequences (45, 46). Our bioinformatic analyses reveal that m^6^A exists both in AAA codons and in iterated A (five or more A’s) stretches found in the coding region of over 80 mRNAs (Table S5). These findings together raise the possibility that one consequence of m^6^A might be to prevent the ribosome from losing frame on consecutive AAA codons.

## Discussion

The elongation of cationic peptides slows the ribosome and can impact the expression of thousands of proteins (Tables S1 and S2) (36, 47). Available evidence suggests that ionic interactions between positively charged peptides and the ribosome account for the reduced translation speeds observed on these sequences (10, 40, 48). We directly tested this model by comparing the rate constants for amino acid incorporation on MK_2_ encoding mRNAs using tRNA^Lys^ aminoacylated with either a positively charged (Lys-tRNA^Lys^) or neutral (Val-tRNA^Lys^) amino acid (Fig. 4). Our investigations reveal that ribosomes translating consecutive AAA codons add a second amino acid (V or K) more slowly. The extent of slowing amino is not dependent on amino acid identity (Val-tRNA^Lys^ k_1_/k_2_ = 6, Lys-tRNA^Lys^ k_1_/k_2_ = 8). In contrast, on consecutive AAG lysine codons, the rate constants for adding the first and second lysine are equivalent (Lys-tRNA^Lys^ k_1_/k_2_ = 1), while a second valine is added substantially more slowly (Val-tRNA^Lys^ k_1_/k_2_ = 20) (Table S3). The inclusion of modifications in tRNA_N_^Lys^ diminished the differences we observed in the rate constants for adding consecutive lysines and valines on AAG and AAA codons (on AAG: Val-tRNA_N_^Lys^ k_1_/k_2_ = 4, Lys-tRNA_N_^Lys^ k_1_/k_2_ = 3; on AAA: Val-tRNA_N_^Lys^ k_1_/k_2_ = 2, Lys-tRNA_N_^Lys^ k_1_/k_2_ = 6) (Tables 2 and S3). Our findings indicate that both peptide charge and codon:tRNA^Lys^ interactions have significant roles in controlling poly(lysine) peptide synthesis rates. These factors play similarly important roles in ribosome frame maintenance during poly(lysine) peptide translation. While we observe ribosome sliding when Val-tRNA^Lys^/Val-tRNA_N_^Lys^ are used (Fig. 4), the rate and extent of sliding on AAA codons are both modestly enhanced by Lys-tRNA^Lys^/Lys-tRNA_N_^Lys^. mRNA sequence (AAA *versus* AAG) and the posttranscriptional modification status of tRNA^Lys^ have larger impacts on frame maintenance than peptide charge. As previously reported, we find that ribosome sliding is only prevalent on lysine encoding mRNAs with consecutive AAA codons (Figs. 3 and S1) (13). We also noticed that the presence of tRNA^Lys^ modifications significantly reduces the rate constant (25-fold) for sliding associated frame loss events (Fig. 3 and Table 2). The strong influence of tRNA_N_^Lys^ modifications on frame maintenance is consistent with previous observations that both bacterial and yeast tRNA with these modifications also promote mRNA–tRNA interactions (34, 49, 50, 51). In addition to enhancing frame maintenance, tRNA_N_^Lys^ modifications appear to be especially important for the addition of valine on AAG, but not AAA, codons by mischarged Val-tRNA^Lys^. The rate constant for Val insertion increases by 70-fold to >1000-fold when fully modified Val-tRNA_N_^Lys^ is reacted on AAG codons (Fig. S8 and Table S3). These findings collectively help to rationalize why loss of tRNA_N_^Lys^ modifications is lethal in yeast and the observation that mutations in the tRNA_N_^Lys^ modification machinery are linked to disease (23, 52, 53, 54, 55, 56).

mRNA–tRNA interactions can be controlled not only by tRNA modifications but also by the posttranscriptional modification of mRNAs. Emerging evidence suggests that mRNA modifications can slow the ribosome and influence the extent of amino acid misincorporation into peptides (22, 57, 58, 59). However, the impact of mRNA modifications on ribosome frame maintenance has not been explored. This question is especially relevant in the context of ribosome sliding because the most common mRNA modification, m^6^A, is present in AAA codons in cells (45, 46). Our analysis of available datasets that map the location of m^6^A transcriptome-wide reveals that m^6^A is included into > 80 mRNAs containing five or more consecutive A’s (Table S5). While m^6^A disrupts both RNA base pairing and tRNA selection by the ribosome, the influence of m^6^A, or any other mRNA modification, on ribosome frame maintenance is not known (42, 60, 61). Our results demonstrate that m^6^A can suppress frame loss events. We find that ribosome sliding is limited when m^6^A is positioned to break up stretches of iterative adenosines (Fig. 5). Together, our biochemical and bioinformatic findings suggest that one consequence of having m^6^A present in these mRNA sequences could be to prevent ribosome sliding in homopolymeric A-rich stretches (62).

While previous studies revealed that the ribosome moves robustly into multiple frames on poly(A) sequences *in vitro*, the mechanism by which this occurs was not known (13, 24, 25). Here, we measured the rate constants for the ribosome moving into five different reading frames while translating an AUG-AAA-AAA-UUC-UAA [MK_2(AAA)_FX] mRNA sequence. These data lead us to propose a model for ribosome sliding in which the ribosome moves backward, one nucleotide at a time, along a homopolymeric(A) sequence until it either encounters an aatRNA^aa^ that it can react with, or enters an unproductive state (Figs. 2 and S3). We find that the rate constants for backward movements by the ribosome are reduced by 50-fold relative to 0-frame amino acid addition and that the greatest levels of nonproductive complex formation are observed following the first −1 movement (Fig. 1*D* and Table 1). The rate constants for these movements, collectively referred to as ribosome sliding, are reduced relative to normal amino acid addition, amino acid misincorporation, and −1 programmed frame shifting (22, 63, 64). While we do observe some amino acid addition in the +1 frame, these events are less robust than their −1 counterparts, and we believe that the +1 frameshifts are not a product of sliding, but rather the result of an empty A site (65).

The slow nature of the progressive −1 movements and formation of nonproductive complexes can help explain why it is possible to capture the ribosome in multiple frames *in vitro* but only visualize ribosome stalling and the first −1 movement in cells. Following the first −1 movement on poly(A) sequence, our results suggest that the ribosome is capable of adding the amino acid in the −1 frame, likely leading to the creation of a prematurely truncated protein product and triggering nonsense-mediated decay. Indeed, there is evidence that nonsense-mediated decay occurs on following −1 frameshifts on poly(A) containing reporters in human cells (14, 66). Additionally, the −1 movement is quite slow (longer than 1 min), giving time for ribosome collisions to occur and a cotranslational quality control mechanism targeting the stalled ribosome complex to be activated (67, 68, 69). Together, this work quantitatively describes how the ribosome translates mRNAs containing poly(A) regions and reveals the important contributions of tRNA–mRNA interactions to ribosome slowing during cationic peptide. Our results suggest that posttranscriptional mRNA modifications may provide cells with a previously unrecognized avenue to ensure that the ribosome remains in the correct mRNA reading frame during the translation of slippery sequences.

## Experimental procedures

### *In vitro* translation assays

70S ICs were prepared using *E. coli* ribosomes programmed with various mRNAs and f-[^35^S]-Met-tRNA^Met^ in the P site (70). Translation was initiated by mixing equal volumes of TC (20–60 μM aatRNA(s), 24 μM EF-G, 60 μM EF-Tu) with ICs (140 nM) in 219-Tris buffer (50 mM Tris pH 7.5, 70 mM NH_4_Cl, 30 mM KCl, 7 mM MgCl_2_, and 5 mM βME). All initiation factors (IF-1, IF-2, and IF-3) and translation factors (EF-Tu and EF-G) used were His-tag purified from *E. coli* using plasmids available from AddGene (26). The reactions were quenched with equal volume of 1 M KOH at discrete time points (0 s–20 min) by hand (5 s–20 min) or using a KinTek RQF-3 quench flow apparatus (0.001–5 s) (71). Each sample was diluted 1:10 in nuclease-free water, and the reactants, intermediates, and products were separated by eTLC, visualized by phosphorimaging, and quantified with ImageQuant (Cytiva Life Sciences) as previously described (26). Depending on the expected peptide products, eTLCs were run in different running buffer conditions to improve separation (26). eTLCs analyzing peptides containing one or more lysines were run in pyridine acetate buffer, pH 2.8, while eTLCs separating peptides with valine (but no lysine) were run in pyridine acetate buffer, pH 5.2. For m^6^A studies in this work, all m^6^A mRNA constructs were purchased from Dharmacon, Horizon Discovery.

### tRNA aminoacylation by synthetases and flexizyme

tRNAs used in experiments were either transcribed with T7 polymerase or were overexpressed and purified from using a pCWAug vector. tRNAs were then aminoacylated using either purified bacterial lysine aatRNA synthetase (LysRS) or misacylated using the dFx flexizyme as described previously (37, 72). In the case of native tRNA_N_^Lys^, a pUC57 plasmid containing the *E. coli* tRNA_N_^Lys^_UUU_ sequence for study was transformed in HB101 cells. These were then grown, purified, and tRNA deacylated for use in synthetase and flexizyme aminoacylation assays, as described previously (26). All other T7 transcribed tRNAs used in this study were aminoacylated using their cognate aatRNA synthetase (AsnRS, IleRS, PheRS, SerRS, and ValRS) and were His-tag purified from *E. coli* using plasmids available from AddGene (26). *E. coli* tRNA_N_^fMet^ was natively purified from a pCWAug vector and methionine was formylated and installed on tRNA_N_^fMet^ using MTF and MetRS enzymes, which were His-tag purified from *E. coli* using plasmids available from AddGene (26, 73).

### Global analysis simulations of amino acid addition

The reactions used to fit and model our data are displayed in Fig. S3. The fits used to obtain *k*_*1*_ and *k*_*2*_ were modeled using differential equations in Kaleidagraph. Subsequent rate constants (*k*_*3+*_) were modeled against simulations using KinTek Explorer. Simulations in KinTek Explorer were run against different potential mechanisms of ribosome sliding (Fig. S3) using data from quantified peptide formation.

### Homopolymeric A sequences in human coding sequences

*Homo sapiens* genome assembly GRCh37 (hg19 release 75, cds.fa) data were used to identify the consecutive A’s in human coding sequences. Consecutive A’s were counted using in-house R scripts. Then, we analyzed single nucleotide resolution m^6^A mapping studies in different tissues (45, 46) to find out whether any of these consecutive A’s have at least one installed m^6^A modification. This analysis yielded m^6^A frequencies that were reported in Table S5.

## Data availability

The authors declare that the data supporting the findings of this study are available within the paper and its supplementary information.

## Supporting information

This article contains supporting information (45, 46).

## Conflict of interest

The authors declare that they have no conflicts of interest with the contents of this article.
