# Supplementary material for: mRNA and tRNA modification states influence ribosome speed and frame maintenance during poly(lysine) peptide synthesis
Source: J Biol Chem. 2022 May 17;298(6):102039. doi: 10.1016/j.jbc.2022.102039 (PMC9207662; doi:10.1016/j.jbc.2022.102039)
Supplement: Supplemental Figures S1–S8 and Tables S3–S5 [file mmc1.pdf]

mRNA and tRNA modification states influence ribosome frame maintenance during poly(lysine) peptide synthesis

Tyler Smith[1], Mehmet Tardu[1], Hem Raj Khatri[1], and Kristin Koutmou[1,2*]

[1]University of Michigan, Department of Chemistry. [2]University of Michigan, Program in Chemical Biology.
*corresponding author

Supplemental Text: **Figures** and **Tables**

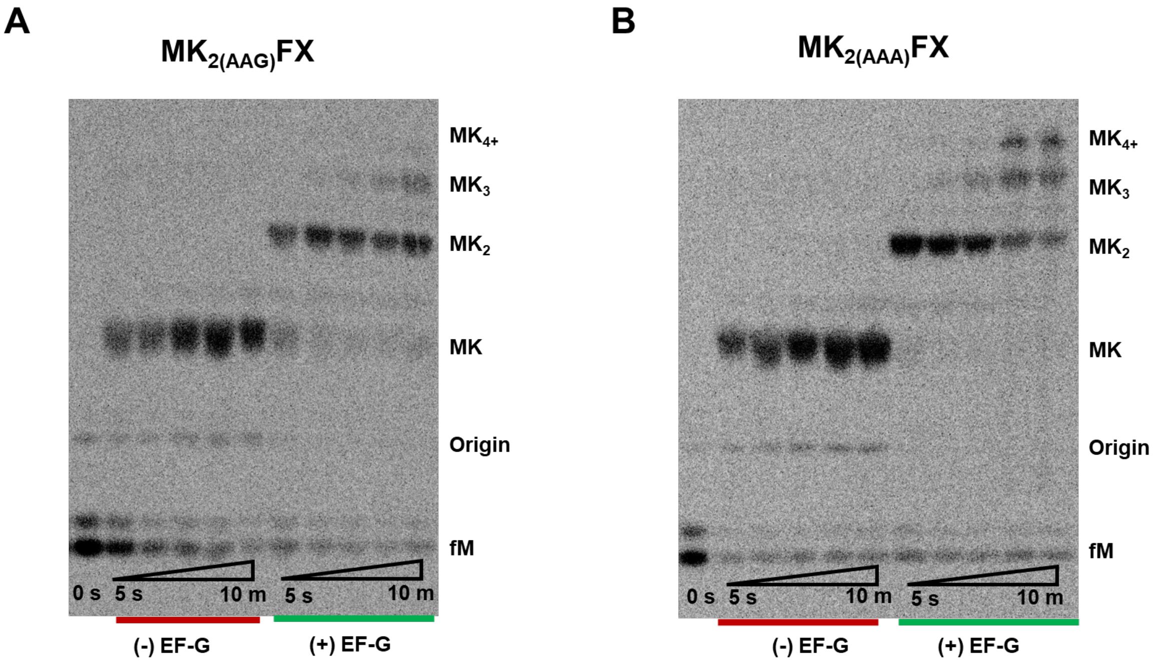

Figure S1 – (**A**) Phosphorimage eTLC of timecourse reactions of lysine addition and ribosome sliding on $MK_{2(AAG)}FX$ mRNA incubated with Lys-$tRNA^{Lys}$ TCs in the absence or presence of translation factor EF-G:GTP. Ribosome sliding, as well as synthesis of products longer than di-peptide, require EF-G:GTP with sliding occurring during steps of translocation. (**B)** Phosphorimage eTLC of timecourse reactions of lysine addition and ribosome sliding on $MK_{2(AAA)}FX$ mRNA incubated with Lys-$tRNA^{Lys}$ TCs in the absence or presence of translation factor EF-G:GTP. Ribosome sliding, as well as synthesis of products longer than di-peptide, require EF-G:GTP with sliding occurring during steps of translocation. In addition, sliding product is formed more robustly compared to AAG codons.

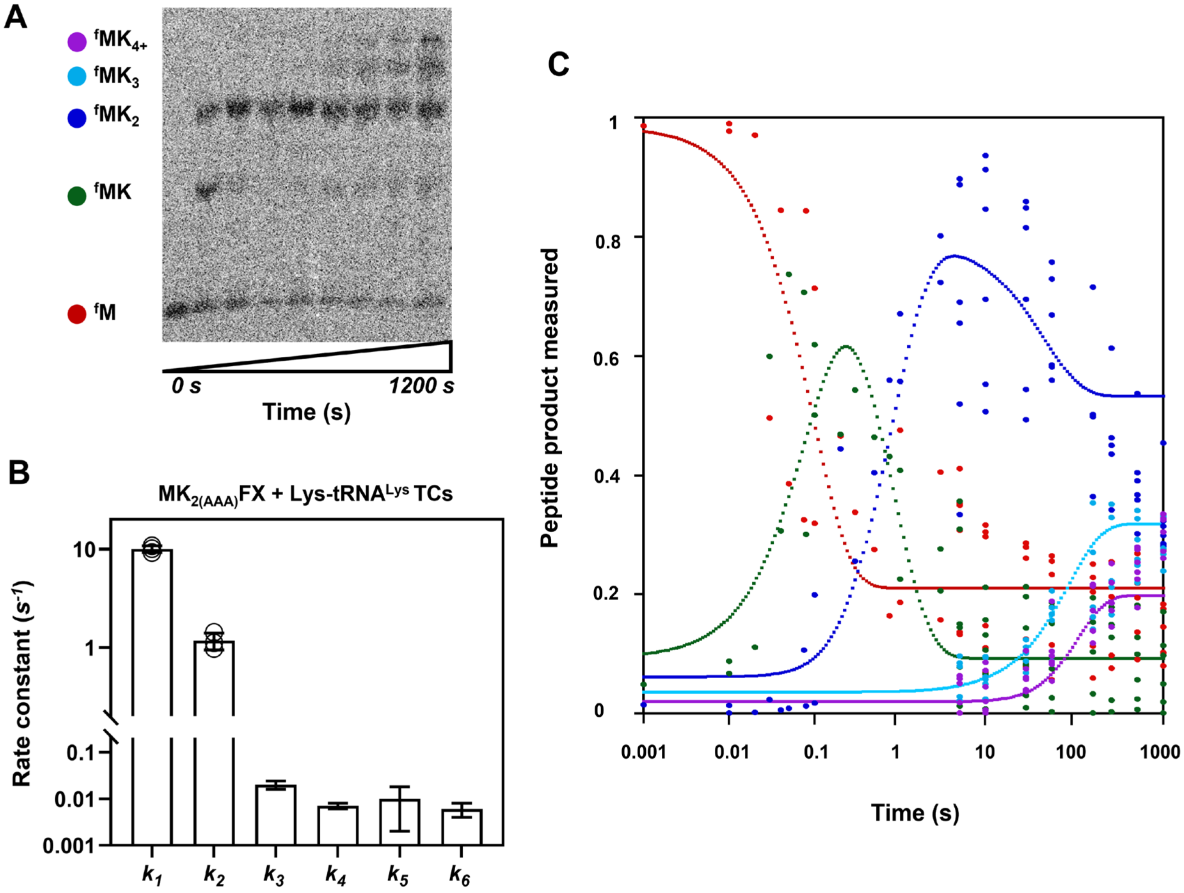

Figure S2 – (**A**) Phosphorimage eTLC of time course reaction of ribosome sliding on $MK_{2(AAA)}FX$ mRNA incubated with Lys-tRNA^{Lys} TCs, as displayed in Figure 3C. Peptide products, indicated by color, were quantified with ImageQuant and then data sets used for global analysis in KinTek Explorer as seen in panel C, with corresponding colors. This eTLC is represented again here as these panels offer a representative flowthrough of the methodology used in the main text from which mechanism and rate constants are derived. (**B**) Rate constants of ribosome sliding on $MK_{2(AAA)}FX$ mRNA using Lys-tRNA^{Lys} TCs (in the presence of EF-G:GTP), as defined by the proposed mechanism presented in Supplemental Scheme 3A. Rate constants for sliding or unproductive ribosome states (k_3, k_4, k_5, and k_6) were obtained via global analysis simulated fitting (as seen in panel C) to determine single rate constants for each step in the proposed Scheme in S3A. Rate constants k_1 and k_2 were obtained by fitting triplicate data sets of ^{f}M disappearance and ^{f}MK formation/disappearance in Kaleidagraph software [$^{f}M = a_1*(1 – (e^{-k_1(t)}) + b$; $^{f}MK = a_1*(1 – (e^{-k_1(t)}) + a_2*(1 – (e^{-k_2(t)}) + b$]. Error bars represent standard deviation. (**C**) Raw data plot time courses (normalized to peptide end-points) of peptide products synthesized during ribosome sliding as exampled in panel A. Global analysis was performed on such data sets to obtain rate constants for lysine addition and ribosome sliding on $MK_{2(AAA)}FX$ mRNA (panel B) as described by the mechanism in Scheme S3A.

A – *Proposed mechanism for ribosome sliding*

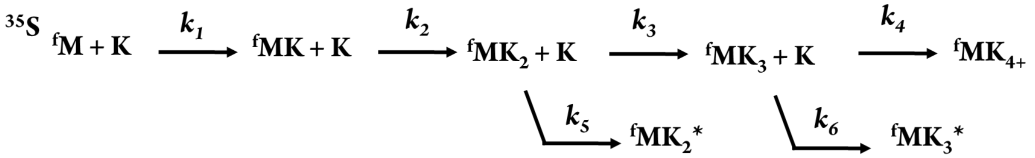

Dipeptide formation: k_1

Tri-peptide formation: k_2

Ribosome sliding and frameshift:

- -3 sliding/frameshift event: k_3, k_4
- Unproductive sliding event(s): k_5, k_6

fMK_2* and fMK_3* represent the first and second unproductive states during sliding, respectfully.

B – *Proposed mechanism for ribosome sliding: Reaction steps with TC substrate concentration subject to consumption*

Dipeptide formation: k_1

Tri-peptide formation: k_2

Ribosome sliding and frameshift:

- -3 sliding/frameshift event: k_3, k_4
- Unproductive sliding event(s): k_5, k_6

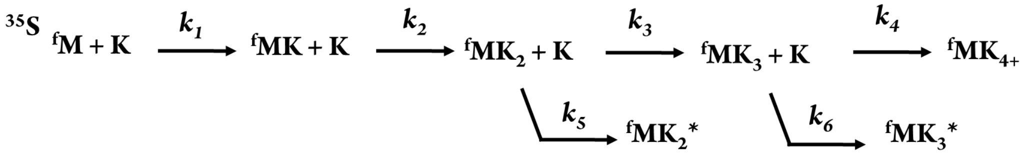

fMK_2* and fMK_3* represent the first and second unproductive states during sliding, respectfully.

Mechanism tested to see if high concentration [30 uM] of Lys-tRNA^{Lys} TCs in assays kept saturating conditions for all lysine addition steps, or if lysine was being consumed more strongly making reaction steps bi-substrate first-order reactions.

C – *Proposed mechanism for ribosome sliding: Pseudo-first order reaction steps with saturating TC substrate (non-productive state formation)*

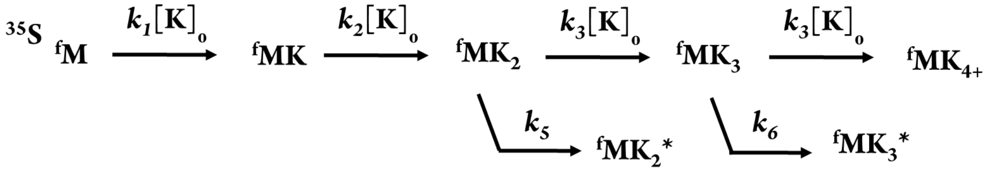

fMK_2* and fMK_3* represent the first and second unproductive states during sliding, respectfully.

Mechanism tested to when high concentration [30 uM] of Lys-tRNA^{Lys} TCs in assays is in fact saturating conditions for all lysine addition steps.

D – *Proposed mechanism for ribosome sliding: Pseudo-first order reaction steps with saturating TC substrate (non-productive state formation)*

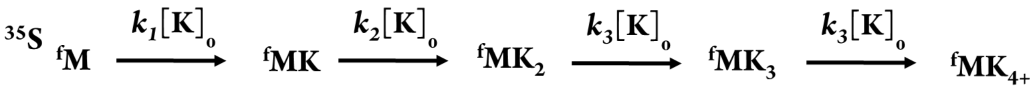

Mechanism tested to when high concentration [30 uM] of Lys-tRNA^{Lys} TCs in assays is in fact saturating conditions for all lysine addition steps, but no non-productive state products are formed.

Figure S3. Tested schemes for ribosome sliding on poly(A) from *in vitro* translation assays. (A-D) These schemes describe tested schemes/mechanisms for subsequent amino acid additions by a ribosome translating on a poly(A) containing mRNA – as displayed in Figure S2. The scheme contains parameters obtainable from the experiments presented here with sets of conditions and parameters used in each scheme tested as detailed above.

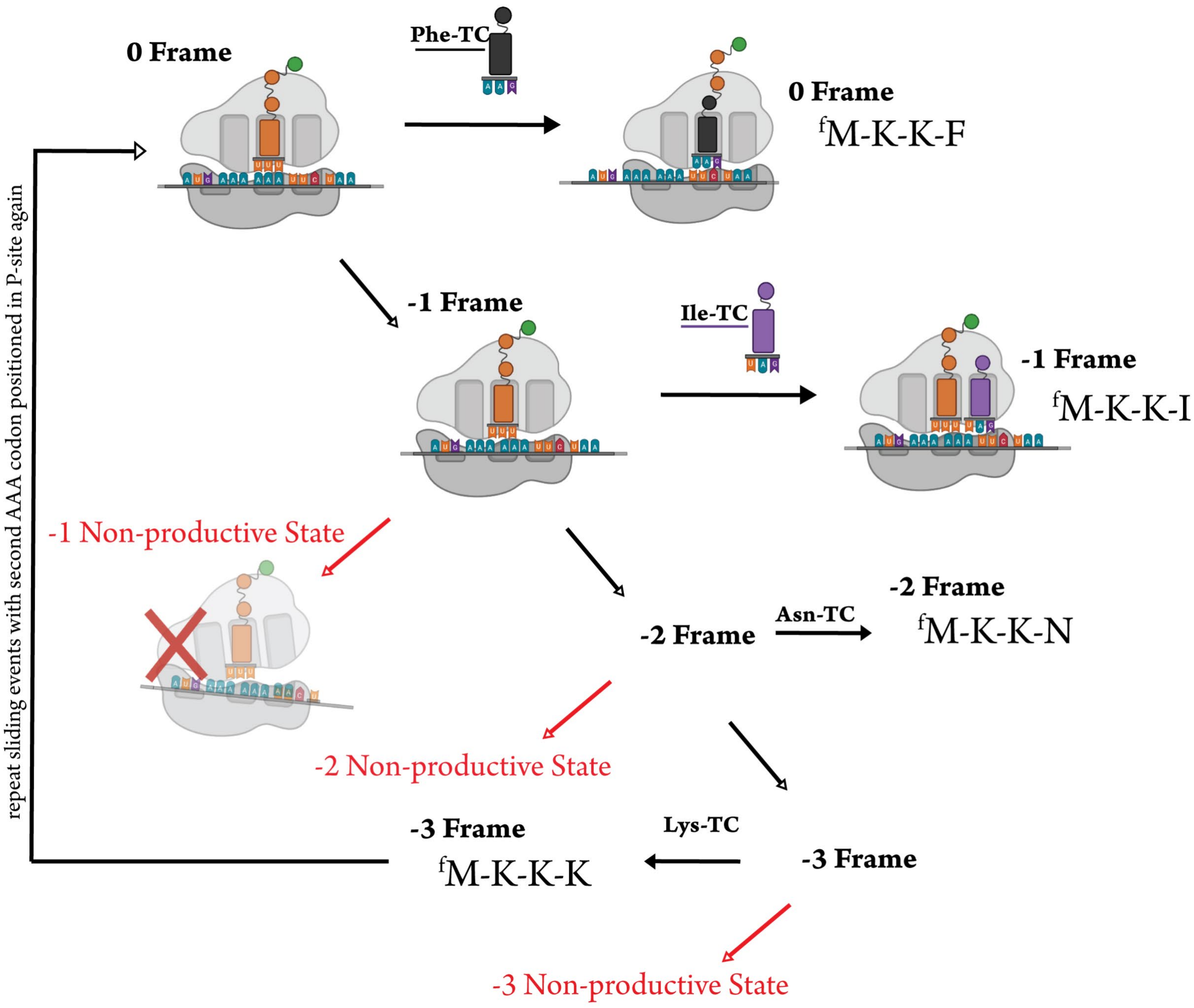

Figure S4 – Scheme of ribosome sliding on poly(A) (example given on $MK_{2(AAA)}FX$ encoding mRNA template). After successfully decoding two AAA codons and incorporating two lysine amino acids, the ribosome is positioned with the next codon in the A-site (UUC). If the cognate amino acyl-tRNA is available (Phe-$tRNA^{Phe}$) it is incorporated, making product in the 0-frame. In the absence of this available aa-$tRNA^{aa}$, the ribosome will shift upstream 1 nucleotide into the -1 frame after a period idling on poly(A) and this will change the identity of the codon in the A site (now AUU). This has the potential to code for a new aa-$tRNA^{aa}$ and, if present, will accommodate the aa-$tRNA^{aa}$ and the ribosome will continue translating in the -1 frame. Similarly, absence of the -1 frame aa-$tRNA^{aa}$ (Ile-$tRNA^{Ile}$) will allow the ribosome to continue moving upstream, in a single nucleotide interval (-2 frame). In addition, the ribosome has the potential to enter a non-productive state during the movement into or from the -1 frame in which the ribosome may enter a rotational state that is unable to accommodate a cognate aa-$tRNA^{aa}$. Similar to the -1 frame event, the ribosome having a now -2 codon in the A-site (now AAU) will code for a new aa-$tRNA^{aa}$ (Asn-$tRNA^{Asn}$) or enter a non-productive state. Once again, if the aa-$tRNA^{aa}$ is absent and the ribosome idles on the poly(A) it will move another nucleotide upstream (now 3 nucleotides, or full codon) in which AAA will be positioned in the A-site. As Lys-$tRNA^{Lys}$ is present in such assays, the ribosome will add another lysine or will enter a non-productive state. If lysine is added in this manner, the ribosome will be in the 0-frame once again and can repeat the cycle as described.

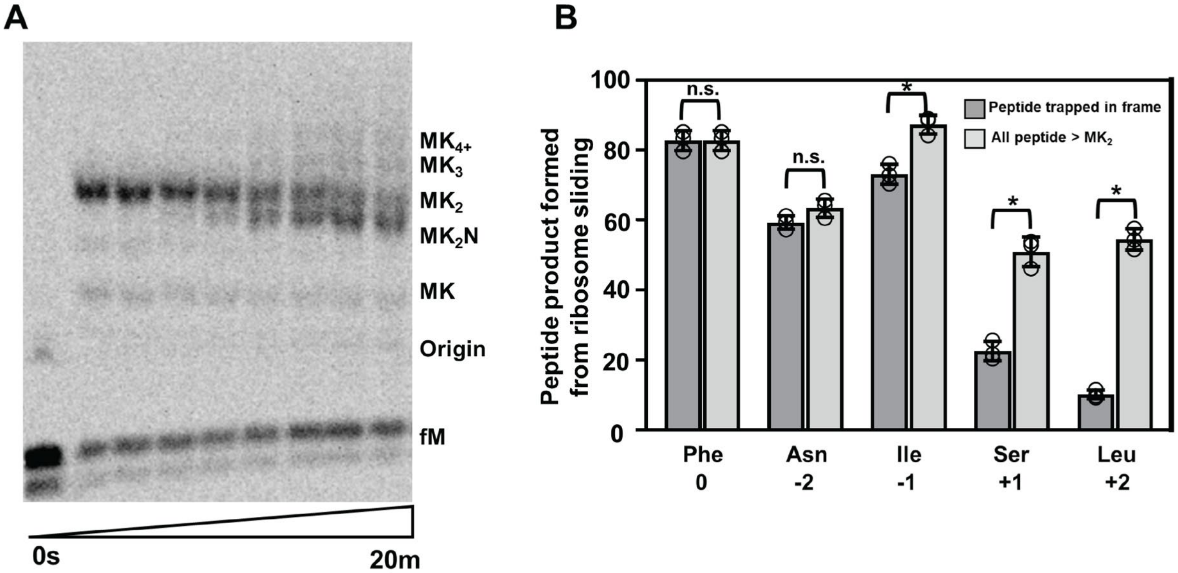

Figure S5 – (**A**) Representative phosphorimage eTLC of time course reaction of ribosome sliding and frameshift on $MK_{2(AAA)}FX$ mRNA incubated with transcribed Lys-$tRNA^{Lys}$ and Asn-$tRNA^{Asn}$ TCs, indicating trapped frameshift products in the -2 frame (MK_2N). (**B**) Each frameshift study reported compared levels of peptide synthesized from frameshifting events to fraction of peptide synthesized from ribosome sliding (greater than tripeptide [MK_2]) from ICs made with $MK_{2(AAA)}FX$ mRNA after 20 minutes. This was done to assess propensity of frameshift into specific frames versus normal amino acid addition or general ribosome sliding on $MK_{2(AAA)}FX$ mRNA. For instance, in the case of the 0 Frame encoding for Phe to form MK_2F tetrapeptide it was observed that ~80% of peptide species synthesized in the assays were longer than MK_2. Of these extended products all peptide synthesized was MK_2F tetrapeptide, indicating normal and efficient Phe addition in the 0 frame. In the case of the -2 frame, adding Asn (as observed in panel A), there was ~62% of sliding peptide product observed versus total peptide species observed ([MK_3 + MK_2N + MK_{4+}] / [MK + MK_2 + MK_3 + MK_2N + MK_{4+}]). However MK_2N was the predominant species synthesized as it comprised ~60% of total peptide product synthesized ([MK_2N] / [MK + MK_2 + MK_3 + MK_2N + MK_{4+}]). This indicates a strong preference to frameshift into, and be trapped in, the -2 frame when Asn-$tRNA^{Asn}$ is available. A similar trend is observed for Ile and the -1 frame, although there is a stronger sliding phenotype observed for -1 frameshifting and sliding. Ser (+1 frame) and Leu (+2 frame) resulted in very little frameshift product as well as extended sliding peptide product. Error bars represent standard deviation. Pairings indicated with a ‘ * ’ represented a significant alteration with a p-value <0.05 using a unpaired student t-test when comparing frame-trapped product against all sliding/frame-trapped product.

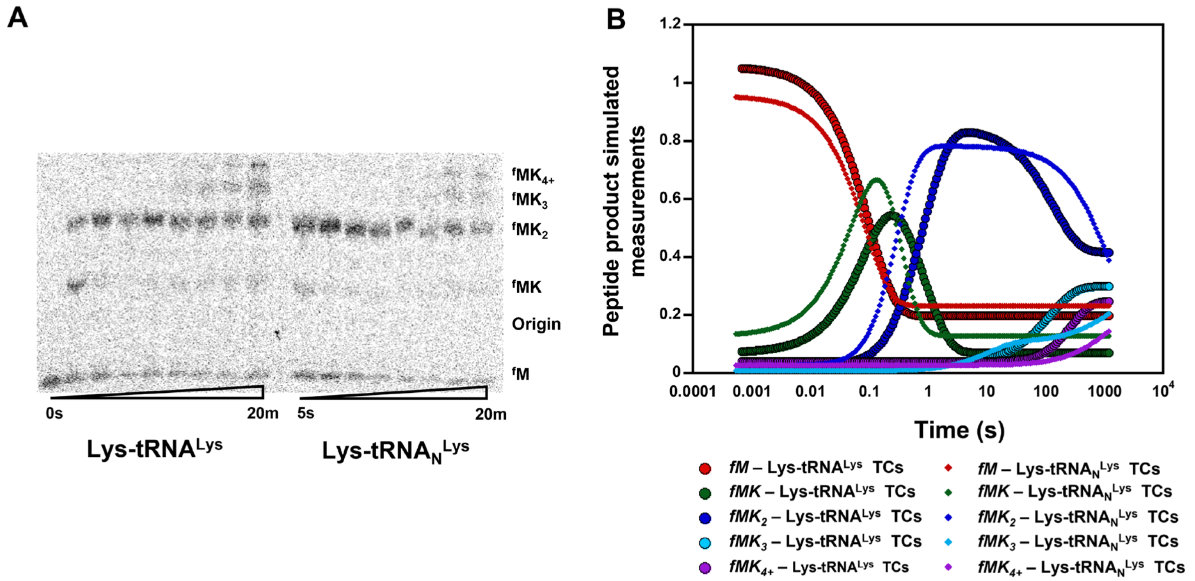

Figure S6 – (**A**) Full representative phosphorimage eTLC of time course reaction of ribosome sliding on $MK_{2(AAA)}FX$ mRNA incubated with Lys-$tRNA^{Lys}$ and Lys-$tRNA_N^{Lys}$ TCs (as shown in Figure 3C and Figure S2A). (**B**) Global analysis was performed on all data sets for assays described in panel A (discussed in methods and Figure S2) and subsequent simulated fits were then overlayed and compared here to view the effect native tRNA modifications have on poly-lysine synthesis when the ribosome reads mRNA containing two iterative AAA codons. Each data set for peptide species was normalized to endpoint (or total peptide product synthesized in the assays) as reflected in the y-axis scale. Synthesis of MK and MK_2 is faster on $MK_{2(AAA)}FX$ when Lys-$tRNA_N^{Lys}$ TCs are used although there is a clear sliding defect with much less MK_3 and MK_{4+} peptide formed compared to when Lys-$tRNA^{Lys}$ TCs are used.

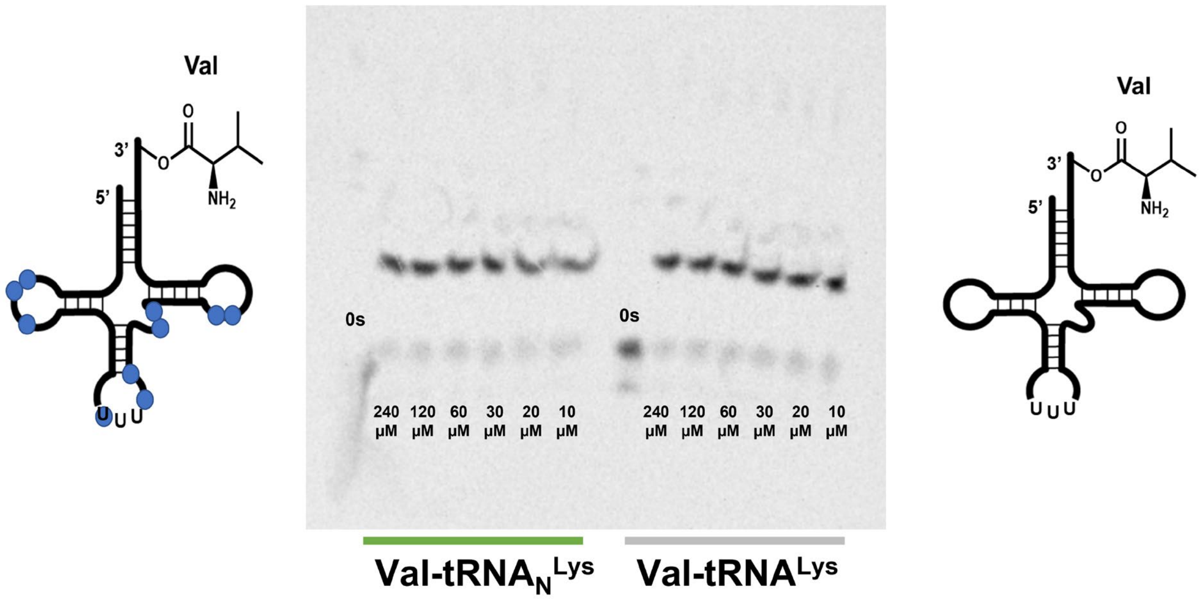

Figure S7 – (**A**) To assess if translation factor EF-Tu:GTP would be selective against incorporating mis-acylated tRNA^{Lys}, translation assays were performed to 5 minute endpoints with varying concentrations of EF-Tu:GTP. Assays used in this main work of this study had final concentrations of 30 uM EF-Tu:GTP which was more than sufficient to incorporate mis-acylated tRNA^{Lys}, regardless of modification status (Val-tRNA^{Lys} on the left representing Native tRNA^{Lys} with example sites in which modified nucleosides are located for E. coli tRNA$^{Lys}_{UUU}$). The phosphorimage eTLC was analyzed using ImageQuant and all samples resulted in at least 88.9% ^{35Sf}Met turnover to synthesize ^{35Sf}Met-Val on a MK$_{2(AAA)}$FX mRNA, regardless of EF-Tu:GTP concentration. These results suggest that EF-Tu:GTP does not select against incorporating Val-tRNA^{Lys} variants in our assays for this study.

A

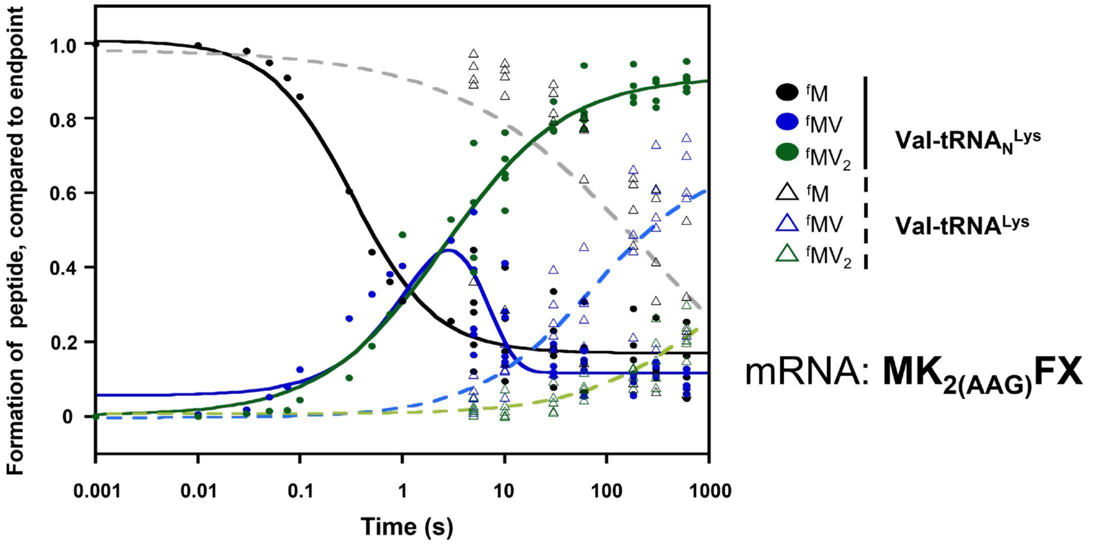

B

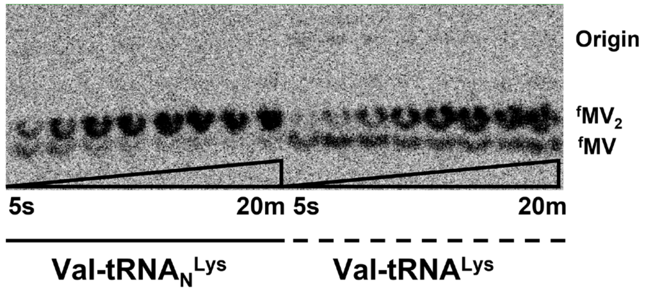

Figure S8 – (A) Analysis performed on all data sets for assays using AUG-AAG-AAG-UUC-UAA mRNA, using either transcribed (Val-tRNA^{Lys}) or native (Val-tRNA$_N^{Lys}$) mis-acylated lysyl TCs. Simulated fits from global analysis are overlaid onto data sets with each tRNA species used, and peptide species formed over time in the translation reactions, indicated. (B) Representative phosphorimage eTLC of time course reaction of valine addition on $MK_{2(AAG)}FX$ mRNA incubated with Val-tRNA^{Lys} or Val-tRNA$_N^{Lys}$ TCs. Lack of modifications to tRNA^{Lys} results in significantly slowed formation of MV and MV_2 peptides from mis-acylated Val-tRNA^{Lys} compared to Val-tRNA$_N^{Lys}$. In addition, as also seen in panel A, there is a drastic decrease in endpoints of di- and tri-peptide products formed when Val-tRNA^{Lys} is used to decode the AAG lysine encoding codon.

Table S3 – Rate constants for valine addition on lysine encoding mRNAs

mRNA construct	$MK_{2(AAA)}FX$		$MK_{2(AAG)}FX$		MV_2FX
**tRNA TCs**	Val-$tRNA^{Lys}$	Val-$tRNA_N^{Lys}$	Val-$tRNA^{Lys}$	Val-$tRNA_N^{Lys}$	Val-$tRNA^{Val}$
**Rate constant (s^{-1})**					
k_1	2.5± 0.3	2.9 ± 0.6	0.06 ± 0.001	4.4 ± 0.07	7.3 ± 0.9
k_2	0.4 ± 0.1	1.7 ± 0.4	0.0003 ± 0.00002	1.2 ± 0.02	1.9 ± 0.4
$k_{3,obs}$	0.002± 0.00005	0.0002 ± 0.00002	-	-	-

**Table S4 – ** Rate constants for lysine addition during ribosome sliding on m^6A containing mRNAs

m^6A containing mRNA	AAm^6A-AAA	AAA-m^6AAA	AAA-Am^6AA	AAA-AAm^6A
**Rate constant (s^{-1})**				
k_1	3.0 ± 0.2	3.2 ± 0.003	11.6 ± 0.8	18.0 ± 2.3
k_2	0.7 ± 0.1	0.2 ± 0.003	0.2 ± 0.003	0.46 ± 0.006
k_3	0.0005 ± 0.0001	0.0006 ± 0.0002	0.0009 ± 0.0003	0.001 ± 0.0001
k_4	0.003 ± 0.0002	0.09 ± 0.02	0.02 ± 0.004	0.1 ± 0.06
k_5	0.0002 ± 0.0001	0.0005 ± 0.0002	0.0007 ± 0.0002	0.001 ± 0.0001
k_6	0.009 ± 0.002	0.1 ± 0.03	0.07 ± 0.02	0.08 ± 0.008

Table S5 – Frequency of m^6A installation in coding sequences‡

Number of consectutive nucleotides (As)	Instances of consecutive Adenosines (A)	Instances with m^6A installed‡‡
4	385832	165
5	125346	60
6	34207	15
7	9237	5
8	2286	2

‡ Frequency of consecutive adenosines as exist in the human hg37.75_cds coding sequence reference genome.

‡‡ Frequency of m^6A installation in consecutive adenosines as reported in human embryonic kidney cells (HEK293) (53) and human liver, brain, and lung tissues (54).

References

45. Linder, B., Grozhik, A. V., Olarerin-George, A. O., Meydan, C., Mason, C. E., and Jaffrey, S. R. (2015) Single-nucleotide resolution mapping of m6A and m6Am throughout the transcriptome. *Nat. Methods*. **12**, 767–772

46. Zhang, Z., Chen, L.-Q., Zhao, Y.-L., Yang, C.-G., Roundtree, I. A., Zhang, Z., Ren, J., Xie, W., He, C., and Luo, G.-Z. (2019) Single-base mapping of m6A by an antibody-independent method. *Sci. Adv.* **5**, eaax0250
